# Unusual Venous-Origin Intraparenchymal Hematoma in the Context of a Post-traumatic Carotid-Cavernous Fistula

**DOI:** 10.7759/cureus.94057

**Published:** 2025-10-07

**Authors:** Diego Páez-Granda, María Magdalena Catota Camacho, Ronald Efraín Barreto Delgado, Edgar Guillermo Ruíz Checa

**Affiliations:** 1 Neuroradiology Section, Hospital Metropolitano, Quito, ECU; 2 Interventional Neuroradiology Section, Hospital Eugenio Espejo, Quito, ECU; 3 Neurology Postgraduate Program, Universidad de las Américas, Quito, ECU; 4 Neurosurgery Department, Hospital de Especialidades Policía Nacional No. 1, Quito, ECU

**Keywords:** carotid artery, carotid-cavernous fistula, cavernous sinus, drainage, fistula, hematoma, intraparenchymal hematoma

## Abstract

Carotid-cavernous fistulas (CCFs) are abnormal connections between the carotid artery and the cavernous sinus. Due to their proximity to the ophthalmic veins, the primary symptoms typically include proptosis, orbital bruit, and chemosis. However, there are a limited number of cases reported in the literature where the clinical presentation is associated with intracerebral hemorrhage.

We present a case of a 59-year-old male who sustained a moderate traumatic brain injury in December 2024, resulting in a right temporal hemorrhagic contusion and encephalomalacia without any clinical sequelae. In February 2025, the patient developed a sudden left hemicranial headache, accompanied by generalized epileptic seizures and altered consciousness, prompting emergency admission. A non-contrast cranial computed tomography (CT) scan revealed an intraparenchymal hematoma in the left anterior lenticular region. A cerebral angiotomography was inconclusive. Given the non-contrast cranial CT scan findings, a cerebral panangiography was performed, which revealed a left CCF. The combination of the clinical history and the imaging findings led to the conclusion of a traumatic origin. The fistula was successfully embolized with coils, and the patient showed neurological improvement during recovery.

Cavernous sinus arteriovenous fistulas present significant diagnostic and therapeutic challenges in clinical practice. The classification of these fistulas into high-flow and low-flow types, according to the Barrow system, provides an initial framework for understanding their nature and clinical presentation. The clinical manifestations can vary depending on the type of fistula, underscoring the importance of understanding the pattern of venous drainage. Endovascular treatment, considered safe and effective, includes options such as coil embolization through either transarterial or transvenous approaches, which are crucial for the management of these conditions. Alternative endovascular treatment options include deploying a flow diverter, though its results in the literature are debatable, and the use of liquid embolic agents. Parent artery sacrifice is reserved as a last resort when selective closure attempts fail.

## Introduction

Carotid-cavernous fistulas (CCFs) are pathological communications between the carotid arterial system and the cavernous sinus, resulting in abnormal shunting of arterial blood into the venous circulation [1]. These lesions are classified by Barrow et al. into direct (high-flow) and indirect (low-flow) types. Direct CCFs (type A) involve a direct connection between the intracavernous internal carotid artery and the cavernous sinus, most commonly following craniofacial trauma or rupture of a cavernous carotid aneurysm [1,2]. Indirect types (B, C, and D) represent dural arteriovenous fistulas formed between meningeal branches of the internal or external carotid arteries and the cavernous sinus, usually occurring spontaneously in elderly or hypertensive patients [2,3]. Less frequent causes include fibromuscular dysplasia, connective tissue disorders such as Ehlers-Danlos syndrome, and iatrogenic injury during neurosurgical or endovascular procedures [4].

The incidence of traumatic CCFs has been reported in up to 2.4%, 8.3%, and 1.7% of anterior fossa fractures, middle fossa fractures, and posterior fossa fractures, respectively [5]. Classically, patients present with ocular manifestations (proptosis, chemosis, orbital bruit, and cranial nerve palsies), resulting from venous hypertension and engorgement of the superior ophthalmic vein [6]. However, the clinical spectrum is broad, and atypical presentations may occur when venous drainage is redirected toward cortical or posterior routes. In such cases, intracerebral or subarachnoid hemorrhage may develop instead of ocular symptoms, a finding rarely reported in the literature [7]. Intracerebral hemorrhage (ICH) as the initial presentation of a CCF is a rare event, with reported associations ranging from 0.9% to 3% [7]. Mori et al. reported a case where a 90-year-old woman presented with ICH due to a non-traumatic direct CCF [8]. The authors concluded that extensive cortical venous reflux was the probable contributor to the hemorrhagic pattern [7].

Recognizing these uncommon variants is challenging, as the absence of typical orbital signs may delay diagnosis and management. We present a case of a traumatic direct CCF manifesting as an intraparenchymal hematoma without ocular symptoms, underscoring the importance of understanding venous drainage patterns in determining clinical presentation and therapeutic approach.

## Case presentation

**Figure 1:**
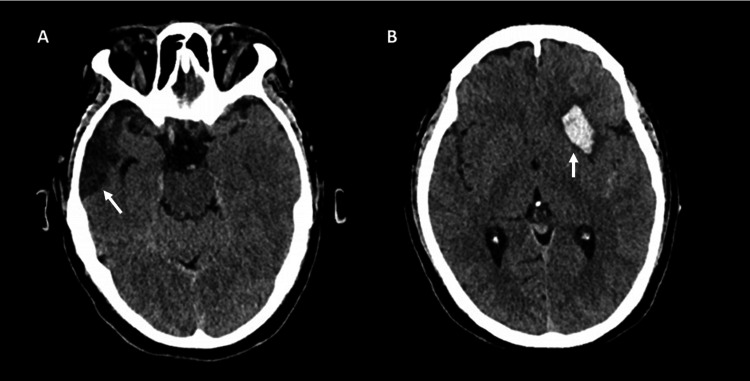
Non-contrast cranial CT scan. A: Encephalomalacia is identified in the right temporal pole (white arrow). B: An intraparenchymal left lenticular hematoma is demonstrated (white arrow).

A 59-year-old male with a history of moderate traumatic brain injury (TBI) was admitted to the ICU in December 2024 for a right temporal hemorrhagic contusion with encephalomalacia, without residual neurological deficits. In February 2025, he developed an acute left-sided headache accompanied by generalized tonic-clonic seizures and a decreased level of consciousness. On arrival, his Glasgow Coma Scale (GCS) score was 9/15. Non-contrast cranial CT revealed the known right temporal encephalomalacia and a new left lenticular intraparenchymal hematoma (Figure 1).

The principal differential diagnosis for a patient presenting with this hemorrhagic pattern was hypertensive intracerebral hemorrhage. This diagnosis was excluded due to the absence of hypertension and the patient's normal arterial blood pressure values. Other differential diagnoses included vascular malformations such as brain arteriovenous malformations (AVMs) and cavernomas.

**Figure 2:**
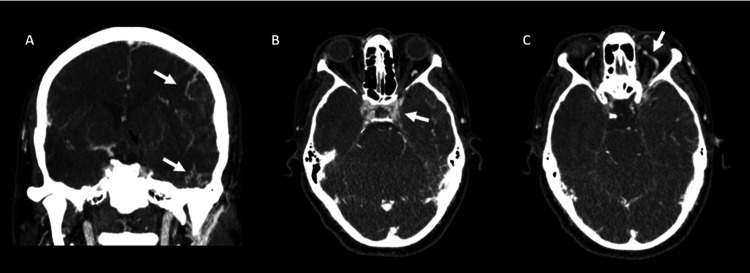
Cerebral angiotomography. A: Tortuous cortical vessels are visualized (white arrows). B and C: Subtle prominence of the cavernous sinus (white arrow in B) and dilation of the supraorbital vein are observed (white arrow in C). No flow in the left internal carotid artery is detected (black arrow in A).

Cerebral CT angiography demonstrated mild dilatation of the left superior ophthalmic vein, subtle prominence of the cavernous sinus, and tortuous cortical vessels (Figure 2), raising suspicion for a vascular lesion.

**Figure 3:**
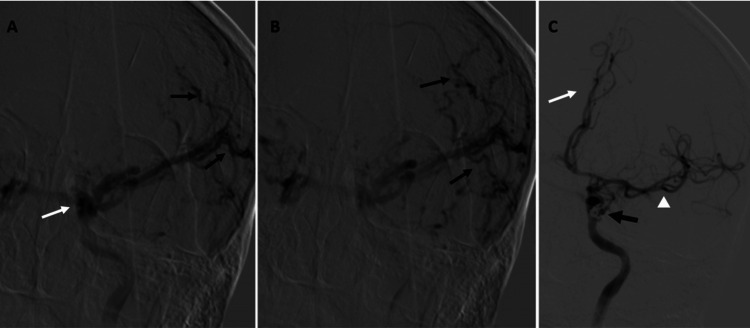
Cerebral panangiography. A and B: Injection through the internal carotid artery reveals the cavernous sinus (white arrow) and tortuous cortical veins (black arrows). C: Post-treatment imaging shows obliteration of the fistula with coils (indicated by the black arrow), along with restoration of vascular flow to the left anterior (white arrow) and middle cerebral arteries (white arrow head).

Subsequent digital subtraction angiography confirmed a direct CCF connecting the left internal carotid artery (ICA) to the left cavernous sinus (Figure 3).

The procedure for endovascular repair of the left CCF was performed under general anesthesia, initiated via a standard right common femoral artery access. The approach utilized a 6 French (6 F) support catheter that was navigated and securely positioned within the left ICA. This placement provided the necessary stable access for the subsequent delivery of embolic material. Selective microcatheterization was achieved across the fistula and into the cavernous sinus. Successful and definitive obliteration of the communication was accomplished through the controlled deployment of two embolization coils. No immediate procedural complications occurred. The patient woke up asymptomatic and remained neurologically intact throughout the following hospital days. Immediate post-procedural angiography confirmed the complete and successful exclusion of the CCF, maintaining excellent antegrade flow through the left ICA (Figure 3). Neurological function improved significantly, and the patient was discharged after a few days of monitoring.

**Figure 4:**
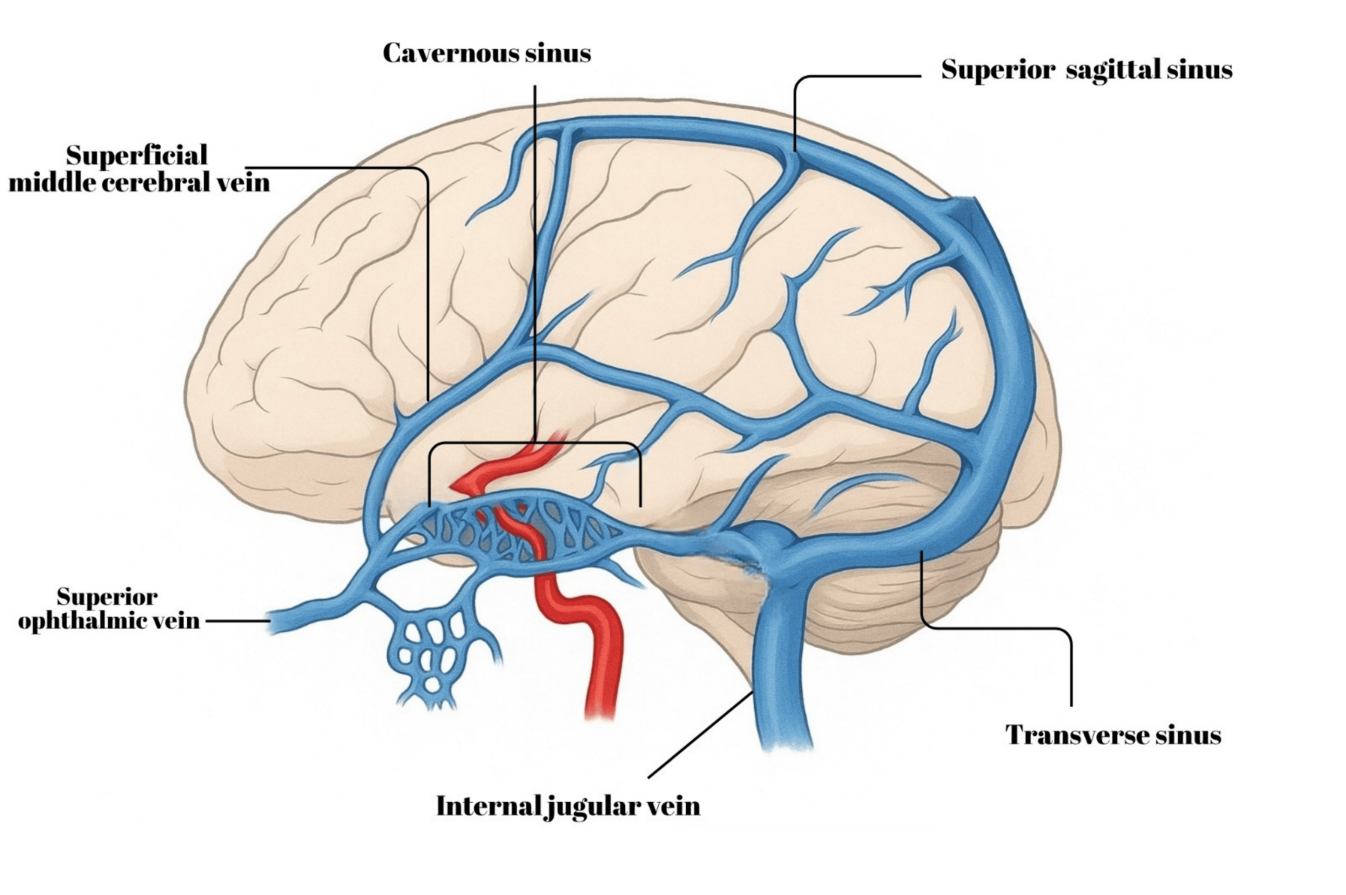
This image demonstrates the relationship between the cerebral veins, particularly the cavernous sinus, and the internal carotid artery. Note the drainage through the superior ophthalmic vein and the superficial middle cerebral veins. Source: Figure adapted from Balcerzak A, Tubbs RS, Zielinska N, Olewnik Ł. Clinical analysis of cavernous sinus anatomy, pathologies, diagnostics, surgical management and complications - Comprehensive review. Ann Anat. 2023 Jan;245:152004. doi: 10.1016/j.aanat.2022.152004. Used under the Creative Commons CC BY-NC-ND 4.0 license.

This case is notable for the absence of typical orbital symptoms, such as proptosis, chemosis, or cranial nerve deficits, with the fistula presenting primarily as an intracerebral hematoma. The atypical presentation underscores the importance of considering traumatic CCF in patients with unexplained intracranial hemorrhage and highlights the role of venous drainage patterns in shaping clinical manifestations (Figure 4).

## Discussion

CCFs represent a complex diagnostic and therapeutic entity, particularly when their presentation deviates from the classical ocular manifestations. In the present case, the CCF was of traumatic origin, most likely resulting from the disruption of the cavernous segment of the ICA secondary to blunt head trauma. Traumatic CCFs are typically high-flow, direct shunts (Barrow type A) and often manifest acutely with orbital signs. However, the absence of such findings in this patient underscores the variability introduced by individual venous drainage patterns.

The occurrence of ICH rather than orbital congestion can be explained by the pattern of venous outflow. Angiographic findings demonstrated predominant cortical venous reflux with minimal anterior drainage through the superior ophthalmic vein (SOV). This configuration leads to intracranial venous hypertension and increases the risk of parenchymal hemorrhage, while sparing the orbit [7]. Although hemorrhagic presentation is extremely uncommon, it has been documented in isolated cases of direct traumatic CCFs with retrograde cortical drainage [7,8]. Recognition of such high-risk venous pathways is crucial for anticipating neurological rather than ophthalmic complications and for tailoring endovascular management accordingly.

Endovascular therapy remains the cornerstone of CCF treatment. In this case, a transarterial approach was selected, appropriate for direct post-traumatic fistulas that communicate with the cavernous segment of the ICA. Transarterial embolization provides rapid and targeted shunt closure while maintaining carotid patency, though arterial access can be technically challenging in complex tears [9]. Transvenous embolization is an alternative when arterial routes are inaccessible or when the fistula drains primarily into the cavernous sinus; however, it carries a higher risk of cranial nerve palsy due to sinus packing [9].

Historically, detachable balloons were widely used in traumatic CCFs for their ability to achieve immediate occlusion and preserve the parent artery, but their use has declined due to availability and migration risks. Liquid embolic agents such as Onyx or NBCA (n-butyl cyanoacrylate) offer controlled delivery and are especially useful in multi-channel fistulas, though they require meticulous technique to prevent reflux [10]. Flow-diverting stents have emerged as a valuable alternative in select direct CCFs, promoting endothelial remodeling and gradual closure of the fistula, though dual antiplatelet therapy and delayed occlusion remain limitations [11]. An individualized approach considering the fistula’s flow dynamics, venous drainage, and need for ICA preservation is essential.

Post-procedural care involves close neurological and angiographic follow-up. Digital subtraction angiography (DSA) or magnetic resonance angiography at three to six months is recommended to confirm durable occlusion and rule out recurrence [12]. Given the initial hemorrhagic presentation, seizure monitoring and prophylaxis are also important. Reported complications include ICA thrombosis, cranial nerve deficits, and recanalization, though overall morbidity remains low with contemporary endovascular techniques.

In summary, this case highlights a traumatic direct CCF with the rare presentation of ICH without ocular signs, attributable to atypical venous drainage favoring cortical reflux. Awareness of such variants is critical for accurate diagnosis, prompt intervention, and prevention of potentially fatal hemorrhagic complications.

## Conclusions

This case underscores the importance of maintaining a high index of suspicion for CCFs, even in the absence of classic orbital manifestations. The unusual presentation with an intraparenchymal hematoma highlights how variations in venous drainage patterns can alter clinical expression and delay diagnosis. Recognizing such atypical forms is essential to prevent neurological deterioration and to guide timely imaging and endovascular treatment. Ultimately, this case reinforces that early identification and appropriate intervention remain critical for achieving optimal neurological and functional outcomes in patients with CCFs.
